# Selection of Soybean Pods by the Stink Bugs, *Nezara viridula* and *Piezodorus guildinii*

**DOI:** 10.1673/031.012.10401

**Published:** 2012-08-27

**Authors:** Gonzalo A. R. Molina, Eduardo V. Trumper

**Affiliations:** EEA INTA Manfredi, Seccion Entomología. Ruta Nacional 9 Km 636, Cordoba, Argentina

**Keywords:** *Glycine max*, food preference, olfactometer, phenological stages, phytophagous, pentatomidae

## Abstract

Different biological parameters of the stink bugs, *Nezara viridula* L. and *Piezodorus guildinii* Westwood (Hemiptera: Pentatomidae), are affected by the developmental stage of the soybean (*Glycine max* Merrill) pods they feed on. These effects of the soybean on the stink bugs could represent a selection pressure leading to the ability of these species to discriminate the phenological stage of soybean pods, and, therefore, to exhibit feeding preferences. We designed three studies: (1) Distant detection of soybean pods through an olfactometer; (2) Free choice tests to evaluate preferences for soybean pods of different developmental stages; (3) No choice tests to study effects of soybean pod development on feeding time and number of probes. Stink bugs showed no differential response to olfactometer arms with or without soybean pods, suggesting an inability to detect soybean volatiles. Free choice tests showed no species effects on pods selection, but significant differences among fifth instar nymphs, adult male, and adult females. Fifth instar nymphs fed more frequently on soybean pods of advanced development stages compared to female adults, despite previous evidence showing poor development of stink bugs fed pods of the same stage. No choice tests showed significant effects of stink bug species, stink bug stage and sex, and soybean pod phenology. *N. viridula* expressed shorter feeding times and higher numbers of probes than *P. guildinii*. The highest numbers of probes of both species were observed when they were fed soybean pods in early phenological stages. When placed in direct contact with food, fifth instar nymphs prefered to feed on more developed pods, despite these pods being suboptimal food items. These results suggest that for the ecological time framework of soybean-stink bugs coexistence, around thirty-five years in Argentina, the selection pressure was not enough for stink bugs to evolve food preferences that match their performance on soybean pods of different development stages.

## Introduction

Most phytophagous pentatomids, commonly referred to as “stink bugs,” are polyphagous, and their wide host range includes both cultivated and wild plants (Panizzi 1997; Coombs 2000). Stink bugs are major agricultural pests across the world (Chocorosqui and Panizzi 2004; Leskey and Hogmire 2005), and they are most often attracted to plants with growing shoots and developing seeds or fruits (McPherson and McPherson 2000; Olson et al. 2011). In Argentina, *Nezara viridula* (L.) and *Piezodorus guildinii* (West.) (Hemiptera: Pentatomidae) are the most important stink bugs, due to their economic impact on soybean production (Massoni and Frana 2005). *P. guildinii* is a neotropical species (Panizzi and Slansky 1985) that feeds on soybean throughout the production areas of Brazil (Oliveira and Panizzi 2003) and Argentina (Massoni and Frana 2005). *N. viridula* originated from eastern Africa, but has been introduced into tropical and subtropical regions of Europe, Asia, Australia, Africa, North and South America (Čokl and Millar 2009). Damage to soybeans is of particular current importance because of recent increases in acreage of this crop in many areas of the world (Čokl and Millar 2009), including Argentina (Ghersa 2005).

Despite their broad host-range, plant use by pentatomids changes with host maturity and phenology, with plants being most attractive during fruit and pod formation stages (Bundy and McPherson 2000). As the fruit/seeds mature, the plants become less attractive, and the stink bugs move to more succulent plants (McPherson and McPherson 2000). They are highly attracted to soybeans during bloom and early pod stages (McPherson et al. 1994), but the highest stink bugs densities generally occur during mid to late pod phenological stages (Musser et al. 2011). In particular, *N. viridula* and *P. guildinii* switch from one host to another, taking advantage of differences in temporal patterns of fruiting in their various hosts (Panizzi 1997). Furthermore, they are known to actively move among soybean fields at different plant phenological stages, even during the reproductive phase (Lourenção et al. 1999; Correa-Ferreira 2005; Gore et al. 2006; Olson et al. 2011). This selective feeding behavior suggests the ability for precise discrimination of food sources, and poses questions regarding the factors that determine foraging decisions.

Optimal foraging theory postulates that animals make decisions that maximize fitness, or a ‘currency’ assumed to be a proxy for fitness, such as rate of energy gain (maximized) or predation risk (minimized) (Stephens and Krebs 1986; Stephens et al. 2006; Raubenheimer et al. 2009). Variation in the quality of food resources has an important effect in the performance of phytophagous insects (Scheirs et al. 2000, 2005). The intrinsic rate of increase of generalist herbivorous insects, particularly plant sucking ones, can vary significantly among host plant species (Panizzi and Slansky 1985; Tsai and Wang 2001; Panizzi et al. 2002; Speight et al. 2008). Developmental and reproductive rates provide important clues concerning the ability of the host to have an impact on fitness. Optimal foraging theory predicts that phytophagous adults should prefer to feed on those host plants or plant organs that confer the highest fitness. This prediction can be applied even to novel plant-insect interactions, as herbivorous insects can evolve adaptations to plants in periods as short as 50 years (Levine and Oloumi-Sadeghi 1996; Schoonhoven et al. 2005). In Argentina, soybean was introduced in the Pampean agricultural region by end of the 1960s (Giorda 1997).

The chemical composition of host plants affects food selection by herbivorous insects. Chemical concentration and composition varies between plant species, and also between plant organs within the same plant (Schoonhoven et al. 2005). Several soybean seed and pod characteristics associated with different phenological stages have positive or negative effects on nymphs and adult performance of stink bugs (Panizzi 1997). In laboratory experiments, Panizzi and Alves (1993) found that *N. viridula* had higher survival, body weight, fecundity, and longevity when they were fed with pods from plants at full seed and initial physiological maturity stages (Fehr and Caviness 1977) compared to other reproductive phenological stages. For *P. guildinii*, Oliveira and Panizzi (2003) found significantly better performance when pods from plants at pod-filling and full seed stages (Fehr and Caviness 1977) were used as food. We hypothesized these significant effects on stink bugs’ development and growth represented a selection pressure to evolve preference ranking for feeding on developmental stages of soybean pods consistent with the performance ranking in life history traits. With this framework, it was questioned whether these species could discriminate soybean pods through volatiles. The benefit of such an ability would contribute to maximizing search efficiency for high quality food sources. Because adults have better dispersal ability than nymphs, we expected them to be more selective.

The following questions were addressed in this study: a) Are *N. viridula* and *P. guildinii* capable of detecting soybean pods through volatiles, and if so, can they discriminate pods at different developmental stages? b) Do these stink bugs show any preference for feeding on soybean pods of specific phenological stages? c) Are these preferences consistent with the stink bugs’ performance ranking stated in the literature, as predicted by optimal foraging theory? d) Does selectivity for soybean pods differ between *N. viridula* and *P. guildinii* and among sexes and insect developmental stages?

Long distance detection of soybean pods by stink bugs was evaluated through olfactometer bioassays, and preferences were assessed through the no choice and free choice experiments.

## Materials and Methods

### Study Area

The study was carried out at Manfredi Agricultural Experimental Station of National Institute for Agricultural Technology (INTA), located in Córdoba province, central Argentina. In order to assure the availability of soybean pods at different development stages, seven soybean plots (10 m^2^) were planted with cultivar DM4600 RR at 10–15 day intervals. Plots were planted following the no tillage method commonly used in the region (Giorda and Baigorri 1997).

### Phenological stages of Soybean.

Soybean development stage system established by Fehr et al. (1971) was used to identify the different stages of soybean developmental phenology. Reproductive stages may be divided into eight classes; R1 and R2 are based in flowering, R3 and R4 on pod development, R5 and R6 on seed development, and R7 and R8 on maturation. We used five stages of maturity: R4 (full pod), R5 (beginning seed), R6 (full seed), R7 (beginning maturity), and R8 (full maturity) (Fehr and Caviness 1977). The study concentrated on reproductive stages of soybeans because the presence of stink bugs on this phase is much more relevant than during vegetative stage (Musser et al. 2011). The highest density levels of stink bugs generally occur during mid to late pod fill (R5–R7) when soybean reproduction stage is advanced (McPherson et al. 1993; McPherson 1996; Bundy and McPherson 2000).

### Pentatomids

Adults of *N. viridula* and *P. guildinii* were collected in soybean fields from Manfredi Agricultural Experimental Station, and kept for at least five days under controlled conditions (25 ± 3° C, 60 ± 9% relative humidity, and photoperiod of 15:9 L:D) for acclimatization. Oviposition was induced by placing female-male pairs in plastic jars (90 × 60 mm) with soybean pods and fresh, daily-renewed leaves. Egg masses were collected and placed in plastic Petri dishes. For better handling and monitoring of stink bugs, groups of five nymphs of the same species were placed in Petri dishes. Approximately 30 grams of fresh soybean pods of each development stage from R3 to R8 were placed in each Petri dish, and were renewed daily until fifth instar nymphs or adults were collected for use in the experiments. Prior to all tests, insects were deprived from food for 24 hours in order to enhance their feeding motivation. Assuming that the feeding behavior of stink bugs can differ on the basis of different physiological demands and abilities to respond to stimuli, three stink bug categories were established: fifth instar nymph, adult female, and adult male.

### Olfactometer assays

An olfactometer was built according to Riddick et al. (2000). Insects (fifth instar nymphs, female adults, and male adults) were placed individually in the main branch of the olfactometer, and 30 grams of soybean pods (R6 for *P. guildinii*, R7 for *N. viridula*) were placed in one of the secondary arms (test arm), while the other secondary arm remained empty as the control (control arm). These phenological stages were selected because they were found to maximize *N. viridula* (Panizzi and Alves 1993) and *P. guildinii* (Oliveira and Panizzi 2003) performance, respectively. Purified air by activated charcoal was blown at a rate of 200 mL/min by a small air pump mounted below the main arm. Time used for acclimation (15 min) of insects in the olfactometer followed Zhang et al. (2007). The amount of time (in min) that each stink bug spent in the test versus the control arm during a 20 min period was recorded. Each test was repeated 20 times per species per stink bug category (Vet and van Opzeeland 1984). The test and control arms of the olfactometer were shifted after every trial. Two olfactometers were used interchangeably.

### Free choice tests

A circular plastic cup was used as the experimental arena (diameter 380 mm). Three groups of soybean pods at phenological stages R4, R5, R6, R7, and R8 (Fehr and Caviness 1977) were distributed at the edges of the container. Each one amounted to 15 g, and comprised 6 to 16 pods depending on the phenological stage. To begin an assay, a single individual was gently introduced into the center of the container with an artist's paint brush (Camel Hair, number 1). Every experiment lasted 30 minutes. The phenological stage of the soybean pod on which the insect chose to feed was recorded. In the cases where an insect changed from one pod stage to another, we recorded the pod phenological stage with the largest time. The species and category of stink bug used in each experimental run were selected at random. Twenty individuals were tested per stink bug physiological maturity stage per species.

### No choice test

In order to evaluate the effects of soybean pods of different developmental stages on feeding behavior, a completely randomized design experiment was carried out with three factors: soybean pod developmental stage (R4 to R8 phenology stages), stink bug category (fifth instar nymphs, adult females, and adult males), and stink bug species, with 10 replicates. Individual test stink bugs were provided with only one of the pod types. Each experimental unit consisted of a Petri dish, in which fifteen grams of pods of one of the development stages were placed in the center, and a single individual that was gently introduced into the center of the container with an artist's paint brush. Each insect was kept in the Petri dish for 60 minutes, during which feeding time, the time the stylets were clearly inserted in the pods (Depieri and Panizzi 2011), was recorded. Also, the number of probes was recorded.

### Statistical analysis

Data obtained with the olfactometer assay were processed with a chi-square contingency table analysis using the software Infostat (Infostat 2009).

In both the free and no choice test, the five developmental stages of soybean pods were grouped into three categories: immature pods (R4 and R5), full seed (R6) pods, and mature (R7 and R8) pods.

Information from the free choice assays consisted of selection frequencies of each soybean pod stage. Because the response variable clearly departs from the standard normality assumption, generalized linear models were used (GLM; McCullagh and Neider 1989). The effects of stink bug category and species on these frequencies were tested with a Generalized Linear Model (Nelder and Wedderburn 1972; Díaz and Demetrio 1998), specifically through a logit model for multi-nomial responses, assuming multinomial distribution of errors (Agresti 2002) represented by the equation
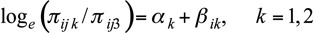

*where π_ijk_* represents the probability of being at level *k* of the polytomous response (categorical variables with more than two values: 1) R4 or R5, 2) R6, 3) R7 or R8) for the *i* category-*j* species combination, *α_k_* is a specific constant for level *k*, and *β_ik_* represents the main effect of category *i* specific for each *k*. In this model, one level of the dependent variable is kept as a reference for comparisons with other levels, and relative risk ratios that represent the change in the odds of being in the dependent variable level versus the comparative level associated with different levels of the categorical explanatory variable are calculated. The *β* coefficients were estimated through maximum likelihood method (McCullagh and Neider 1989).

Data from the no choice test were analyzed with a log-linear model through a Generalized Linear Model with logarithmic link (Scheiner and Gurevitch 1993). Poisson and Negative Binomial models were evaluated for taking into account the distribution of errors. Analysis was performed using the statistical software package R (R Development Core Team 2010), and selection of best statistical models was performed through likelihood ratio test. As a complement to this analysis, the Kullback-Leibler Information method was applied (Burnham and Anderson 2002). Taking into account the Akaike Information Criterium (AIC) values for each model and calculating the AIC differences (Δ_i_), a clear model ranking can be established, with the lowest AIC (Δ_i_ =0) being the criterion for identifying the best single model. According to Burnham and Anderson (2002), other candidate equations having Δ_i_ values within 2 of the best model can be considered to have substantial support.

## Results

In the olfactometer experiment, the observed frequency of positive responses (selection of the olfactometer arm with soybean pods) did not differ from a random distribution with any of the stink bug categories either in *N. viridula* (X^2^ Pearson = 0.54; df = 2; *p* = 0.7650; Table 1) or *P. guildinii* (X^2^ = 2.31; df = 2; *p* = 0.3147; Table 1). Similarly, pooling data from both species revealed that the frequency of positive responses did not differ from a random distribution (X^2^ = 2.49; df = 2; *p* = 0.2874; Table 1).

Visual examination of the free choice test results suggests that the pattern of pod selection differed among nymphs, adult females, and adult males, and that insect category had a statistically significant effect. The deviance change when a model with stink bug category was compared to a null model was significant (deviance = 10.8; d.f. = 4; P = 0.028; Table 2). When stink bug species was incorporated as a second factor, the change in deviance was not significant (deviance = 11.01; df = 6; *p* = 0.088; Table 2). Consequently, the logit model for multi-nomial responses with stink bug category as the only factor was the most appropriate. Nymphal stage was set as the reference, and was compared with the females and males categories. A close examination of this model (Table 3) shows that, with data combined over the two species, the chance of a stink bug selecting and staying on mature pods (R7 and R8) instead of full seed pods (R6) was 5.2 times higher (*p* = 0.006) in fifth instar nymphs than in adult females. Also, the chance of a stink bug selecting mature pods (R7 and R8) instead of immature pods (R4 and R5) was 2.8 and 2.6 times higher in fifth instar nymphs than in adult females and adult males, respectively, at marginal significance levels (*p* = 0.07). A summary of data from the free choice test is found in Table 4.

**Figure 1.**
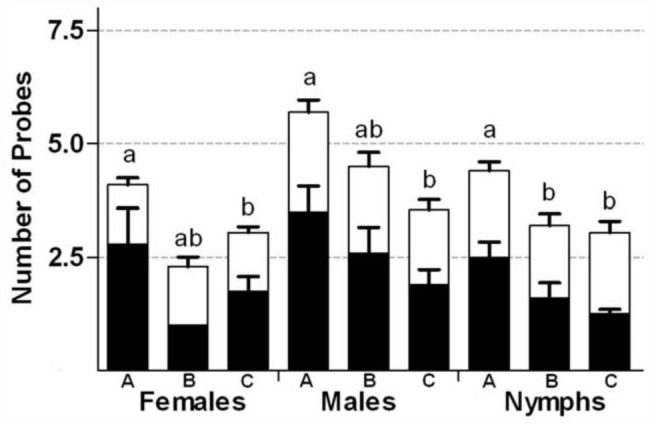
Mean number of probes in 60 minutes for *Nezara viridula* (black bars), and *Piezodorus guildinii* (white bars), recorded on each pod phenological group ((A: R4 and R5, B: R6, and C: R7 and R8), arranged by stink bug categories. Significant difference (P < 0.002) was found for Stink bugs species and for stink bug category (P < 0.02). Means values (bars) and SE (whiskers). Phenological groups within stink bug categories with different letters are significantly different (glm test, P < 0.05). High quality figures are available online.

Log-linear modelling analysis applied to no choice test results required that the random component be modeled through a negative binomial distribution, which significantly improved the statistical fit compared to the Poisson distribution. This analysis showed that number of feeding probes was affected by developmental stage of the soybean pods, stink bug category, and stink bug species. Interaction terms were not significant (Table 5). Model ranking based on the AIC differences (Δ_i_) shows that the best model was the one including the three main factors with no interactions. Models with interaction terms do not warrant consideration, because they yielded Δ_i_ values very near or even greater than 2, beyond the range of “substantial support” suggested by Burnham and Anderson (2002). Thus, each main factor can be analyzed independently. For the number of probes, differences were found between immature and full seed pods (*p* < 0.003), and between immature pods and mature pods (*p* < 0.0001), with larger values for immature pods. Nymphs and females showed higher number of probes than males (*p* < 0.016 and *p* < 0.002, respectively; Figure 1). In addition, *N. viridula* had higher number of probes than *P. guildinii* (*p* < 0.002; Figure 1).

**Figure 2.**
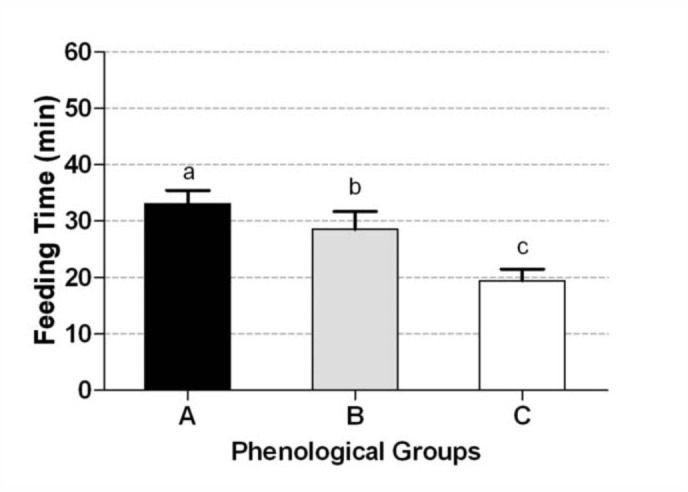
Mean feeding time in 60 minutes in no choice test for phenological group A (black bar), phenological group B (grey bar), and phenological group C (white bar). Data from *Nezara viridula* and *Piezodorus guildinii* were pooled together. Means values (bars) and SE (whiskers) are shown. Phenological groups (A: R4 and R5, B: R6, and C: R7 and R8) with different letters are significantly different (glm test, *p* < 0.05). High quality figures are available online.

On the other hand, among the models fitted to describe variation in feeding time, the likelihood ratio test showed that the best model included soybean pod stage as the only explanatory factor (Table 6). According to model ranking based on AIC differences, two subsequent models that only included main factors with no interaction terms had Δi values within 2 of the best model (Table 7). Considering that no interaction terms were warranted in the selected models to describe variation of feeding time, it was appropriate to consider the model with only soybean pod phenological group factor. Feeding time in mature pods was lower than in immature pods (*p* < 0.001) and full seed pods (*p* < 0.042; Figure 2), without a detectable difference between immature pods and full seed pods.

## Discussion

Olfactometer assays showed no evidence in support of the hypothesis that *N. viridula* and *P. guildinii* can detect and discriminate their food following volatile cues emitted by food source, even though the experiments used soybean pods at developmental stages that were demonstrated to best meet nutritional requirements of these stink bugs (Panizzi and Alves 1993; Oliveira and Panizzi 2003). However, it is not possible to determine whether soybean pods do not release volatiles at all, or whether the concentration of volatiles is too low for detection by stink bugs. In a study conducted with the stink bug *Eurydema pulchrum* Westwood on intact and macerated plants, Rather et al. (2010) showed distant attraction with higher response to macerated plants related to an increase of volatiles concentration when plant cells were crushed or injured. Although macerated plant tissues could enhance attraction of herbivore insects, this is not a normal stimulus for stink bugs in soybean fields.

Panizzi et al. (2004), working with sub-fractions of a chemical extract from soybean pods, found a change in oviposition behavior under the presence of certain compound blends, proving that stink bugs indeed are able to detect them. Although oviposition does not necessarily relate directly with adult feeding, the preference-performance hypothesis postulates a positive relationship to offspring performance by choosing oviposition substrates that provide better food for immature stink bugs stages. These results using contact compounds would indicate a certain level of specificity over the blend compounds of the host. Possibly, these stink bugs are able to detect their hosts at a distance. Our olfactometer results indicate that stink bugs are not able to detect soybean pods at a distance, but other tissues may be involved in the release of volatile compounds. The lack of a specific mechanism in *N. viridula* and *P. guildinii* for directly detecting the most appropriate food source could stem from flexible feeding habits. This argument is more consistent for *N. viridula* given its wide host range compared to *P. guildinii*, which is largely restricted to Fabaceae. *N. viridula* is highly polyphagous, feeding on both monocotyledonous and dicotyledonous plants from more than 30 families, with a distinct preference for legumes (Todd and Herzog 1980; Panizzi and Slansky 1991; Panizzi 2000; Panizzi et al. 2000). As with other multivoltine stink bugs, *N. viridula* switches from one host to another, taking advantage of differences in temporal patterns of fruiting in their various hosts (Panizzi 1997). Plants with developing fruits or pods appear to be more attractive than those with mature ones (McPherson and McPherson 2000).

In the free choice tests, in which all developmental stages of soybean pods were available to experimental insects, fifth instar nymphs showed a stronger attraction to mature stages (R7 and R8) of soybean pods compared to adult females. Panizzi and Alves (1993) found high mortality of nymphs fed pods in phenological stage R8, which can be related to the hardness of the pods’ walls, as well as their hairiness. Our results indicate that the pod stages more preferred by the nymphs (mature stages) give the worst values of performance. Feeding on these pod stages negatively affects adult survivorship, weight gain, lipid content, and reproductive performance, and only 30–40% of the nymphs reached adulthood (Panizzi and Alves 1993; Oliveira and Panizzi 2003). Other factors must have had a stronger attractive effect. Most diurnal insects are attracted by the color yellow (for example, Lepidoptera, Diptera, and Homoptera; Schoonhoven et al. 2005). In many cases yellow surfaces act as “super-normal” stimulus, because they emit an energy peak in the same band width as foliage, but at a higher intensity (Schoonhoven et al. 2005). Soybean pods in R8 phenological stage are yellowish, so this could explain why nymphs preferred them over other stages. No evidence of such hierarchy of visual stimuli in stink bugs is available in literature, but Patt and Sétamou (2007) found that *Homalodisca coagulata* Say nymphs were strongly attracted by yellow stimuli without regard to chemical stimuli.

In the no choice test, the feeding time in both stink bug species was similar, in agreement with Depieri and Panizzi (2011). Regardless of species and stink bug category, feeding times were able to be differentiated according to the phenological stage of soybean pods. The fact that stink bugs fed for longer time on immature and full seed pods (R4 to R6) than on mature pods (R7 and R8) suggests that they are able to assess the quality of food substrates (Simpson 1982). Feeding behavior can be affected by chemical and physical characteristics of the food substrate. Mature soybean pods contain lower concentrations of nitrogen (Egli and Bruening 2007), have tissues more resistant to mechanical damage, and have a dense pilosity (Panizzi and Oliveira 2003) due to thickening of cell walls and a higher proportion of lignin (Capeleti et al. 2005, Saes Zobiole et al. 2010). Obermaier and Zwölfer (1999) showed that *Oreina luctuosa* can overcome low levels of nitrogen either by selecting younger leaves with higher nitrogen concentrations, or by increasing the daily food consumption rate on leaves with a low level of nitrogen by a prolongation of the feeding period. However, in our study, stink bugs fed for longer periods on pods with higher nitrogen concentrations. Physical resistance characteristics prevent injuries caused by herbivores, so the number of probes should be expected to increase in mature pods in order to obtain the nutrients for adequate development. Our study showed a longer feeding time on immature pods than on mature ones. Mature pods have dense pilosity that inhibits stink bug movement, and the insects may be forced to walk. If this difficulty is significant, stink bugs would be forced to walk around over the pods surface until they find an appropriate spot for making a probe. High investment of time preparing for feeding might reduce the available time for the rest of the feeding process, hence the lower values of feeding time and numbers of probes in mature pods.

Differences between nymphs and female adults compared to male adults could be explained by different nutritional requirements among them. Nutrient requirements are higher for the successful development of nymphs and successful reproduction by adult females (Lee 2010). Piubelli et al. (2003) established that female adults of *N. viridula* accumulate greater lipid content than male adults, which suggests higher metabolic demands, and possibly longer feeding times. In addition, *N. viridula* made more probes than *P. guildinii*. This could be explained because the polyphagous habit of *N. viridula* would make them more easily affected by external stimuli, particularly volatiles from potential food sources (Bernays et al. 2004). Polyphagous insects respond to a wider array of stimuli than monophagous insects, and this creates greater “processing” demand on the polyphagous insects, which leads to longer “decision-making” intervals (Bernays 1999; Bernays and Funk 1999). Food selection can take a considerable time due to the competition among inputs.

Previous studies (Panizzi and Alves 1993; Oliveira and Panizzi 2003) show that feeding on soybean pods in the phenological stages R7 and R6 by *N. viridula* and *P. guildinii*, respectively, increase their performance. Our study showed no preference by the stink bugs for these phenological stages. More specifically, the preference for mature pods in nymphs does not occur in adults. Because nymphs have lower dispersal ability to search for better food sources compared to adults, they may be less flexible in the selection of available food. Thus, nymphal food sources may depend on maternal choice of oviposition sites. Selection of a host in the mature stage by stink bug females affects their offspring, because the probability of laying eggs on them increases, in which case offspring would have low performance (Panizzi and Alves 1993). In this context, individual performance conflicts with offspring performance. Thus, non-preference for mature pods could be interpreted as a tendency to avoid an inappropriate substrate for the progeny, particularly younger instar nymphs. Optimal oviposition theory (Jaenike 1978; Price 1997), which postulates a strategy for selecting substrates for egg laying that maximizes the offspring performance, supports this interpretation.

In our study, it was found that soybean pods did not attract stink bugs. It remains unclear whether this actually reflects non-long-distance attraction, whether soybean pods release plant volatiles in concentrations too low to be detected by pentatomids. Ranking of preferences showed by *N. viridula* and *P. guildinii* for phenological stages of soybean pods did not clearly match with their differential performance on them (Panizzi and Alves 1993; Oliveira and Panizzi 2003). Consequently, it was concluded that either the selection pressure was not as strong as hypothesized, or there has not been enough time of soybean-stink bug interaction for evolving a specific food selective behavior.

**Table 1.**
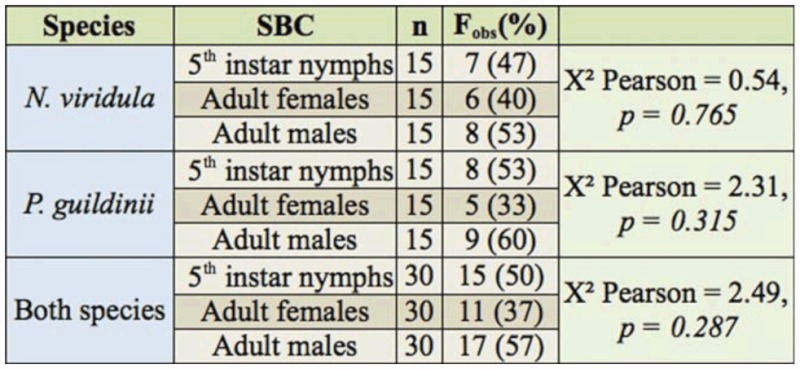
Attraction of soybean pods to stink bugs of different categories (SBC) in olfactometer bioassays. Soybean pods were used at phenological stages R6 for *Piezodorus guildinii* and R7 for *Nezara viridula* (Panizzi and Alves 1993; Oliveira and Panizzi 2003). Analysis was based on contingency table. The variable F_obs_ are the positive cases with percentage in parentheses.

**Table 2.**
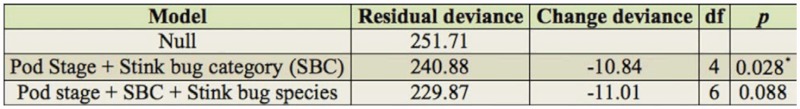
Analysis of deviance for the chance of stink bugs selecting soybean pods of different developmental stages. Analysis was based on Generalized Linear Models with binomial errors and logit link using maximum likelihood estimation. ^*^ Significance of treatment effect.

**Table 3.**
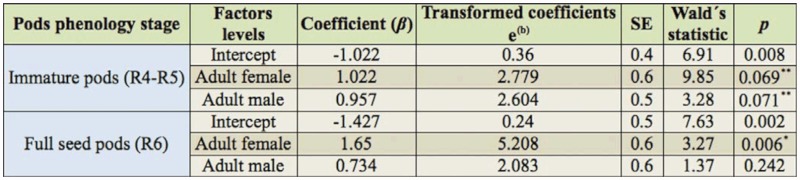
Generalized linear model for the chance of stink bugs selecting soybean pods of different developmental stages. Analysis was based on the selected GLM with binomial errors and logit link using maximum likelihood estimation. Effects of factor levels were tested with Wald tests. Mature pods and fifth instar nymphs of the multinomial response variable served as the reference category. ^*^Significance of treatment effect. ^**^Marginal significance of treatment effect.

**Table 4.**
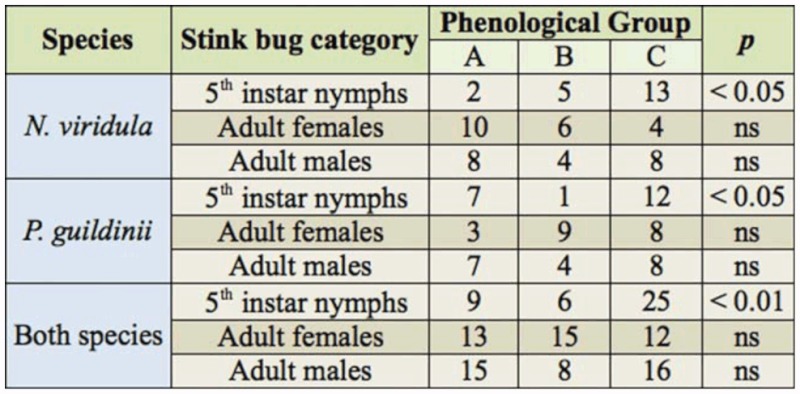
Number of individuals selecting different phenological groups (A: R4 and R5, B: R6, and C: R7 and R8) of soybean pods recorded in a free choice test.

**Table 5.**
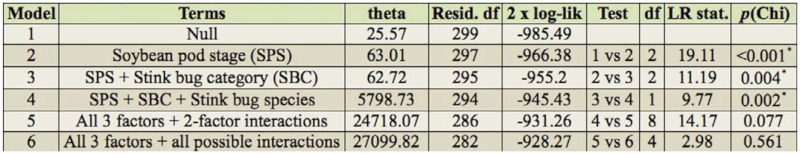
Likelihood ratio test of negative binomial generalized linear model for number of probes. Analysis was based on the selected GLM with binomial errors. Phenological stages of pods were pooled in groups (immature pods, full seed pods, and mature pods). ^*^ Significance of treatment effect.

**Table 6.**
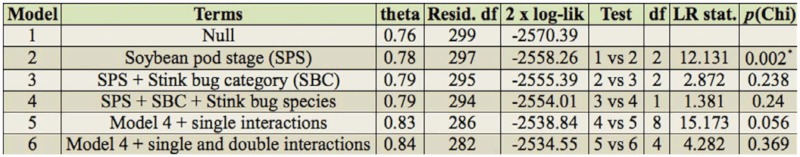
Likelihood ratio test of negative binomial generalized linear model for feeding time. Analysis was based on the selected GLM with binomial errors. Phenological stages of pods were pooled in groups (immature pods, full seed pods, and mature pods). ^*^ Significance of treatment effect.

**Table 7.**
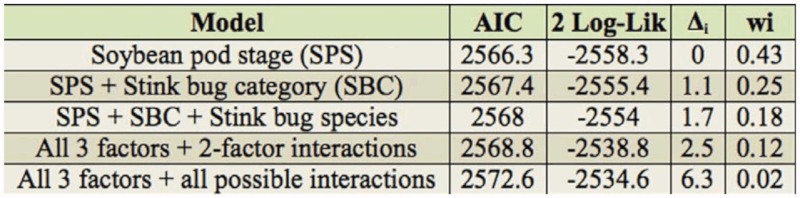
Akaike Information Criterium (AIC) values for each model for the variable Feeding time. Values of AIC differences (Δ_i_) within 2 of the best model, can be considered to have substantial support.
